# Eco-anxiety: a new disease or a “new normal”?

**DOI:** 10.47626/2237-6089-2022-0543

**Published:** 2024-01-08

**Authors:** Enrique Falceto de Barros, Olga Garcia Falceto, Rafaela Brugalli Zandavalli, Diogo Onofre Souza

**Affiliations:** 1 Programa de Pós-Graduação em Educação em Ciências: Química da Vida e Saúde Universidade Federal do Rio Grande do Sul Porto Alegre RS Brazil Programa de Pós-Graduação em Educação em Ciências: Química da Vida e Saúde , Universidade Federal do Rio Grande do Sul (UFRGS), Porto Alegre , RS , Brazil .; 2 Departamento de Psiquiatria e Medicina Legal UFRGS Porto Alegre RS Brasil Departamento de Psiquiatria e Medicina Legal , UFRGS , Porto Alegre , RS , Brasil .; 3 Grupo Hospitalar Conceição Porto Alegre RS Brasil Medicina de Família e Comunidade, Grupo Hospitalar Conceição , Porto Alegre , RS , Brasil .

A cutting-edge theme for mental health disciplines, as described in a recent letter in The Lancet Planetary Health, is the pathologization of climate and eco-anxiety. ^1^ There is a growing awareness that our hyperconsumption, inequitable, and fossil fuel addicted civilization is making our home planet sick. In an international survey of 10 thousand children and young people across 10 countries including Brazil, up to 59% of children and young people were reported as very or extremely worried about climate change. More than 45% of respondents said their feelings about climate change negatively affected their daily life and functioning. ^2^ There is no doubt we are experiencing a psychological shift in our civilization. What should clinicians do about it? One possible roadmap is offered by the new field of Planetary Health (PH). PH studies anthropogenic effects on the environment and their negative feedback on human health, while also offering evidence-based pathways to a healthier civilization and home planet. The São Paulo Declaration on Planetary Health (Declaração de São Paulo sobre Saúde Planetária) makes recommendations for the healthcare sector to lead our global healing. ^3^ Psychiatrists must join this debate and meditate about whether eco-anxiety is a new disease or a “new normal.”
